# Defining novel causal SNPs and linked phenotypes at melanoma-associated loci

**DOI:** 10.1093/hmg/ddac074

**Published:** 2022-03-31

**Authors:** Carolina Castaneda-Garcia, Vivek Iyer, Jérémie Nsengimana, Adam Trower, Alastair Droop, Kevin M Brown, Jiyeon Choi, Tongwu Zhang, Mark Harland, Julia A Newton-Bishop, D Timothy Bishop, David J Adams, Mark M Iles, Carla Daniela Robles-Espinoza

**Affiliations:** Laboratorio Internacional de Investigación sobre el Genoma Humano, Universidad Nacional Autónoma de México, Santiago de Querétaro, México 76230, USA; Cancer, Ageing and Somatic Mutation, Wellcome Sanger Institute, Hinxton, Cambridgeshire CB101SA, UK; Biostatistics Research Group, Population Health Sciences Institute, Faculty of Medical Sciences, Newcastle University, Newcastle upon Tyne NE2 4BN, UK; Leeds Institute of Medical Research, School of Medicine, University of Leeds, Leeds LS9 7TF, UK; Leeds Institute for Data Analytics, University of Leeds, Leeds LS9 7TF, USA; Cancer, Ageing and Somatic Mutation, Wellcome Sanger Institute, Hinxton, Cambridgeshire CB101SA, UK; Division of Cancer Epidemiology and Genetics, National Cancer Institute, National Institutes of Health, Bethesda, MD 20892, USA; Division of Cancer Epidemiology and Genetics, National Cancer Institute, National Institutes of Health, Bethesda, MD 20892, USA; Division of Cancer Epidemiology and Genetics, National Cancer Institute, National Institutes of Health, Bethesda, MD 20892, USA; Leeds Institute of Medical Research, School of Medicine, University of Leeds, Leeds LS9 7TF, UK; Leeds Institute of Medical Research, School of Medicine, University of Leeds, Leeds LS9 7TF, UK; Leeds Institute of Medical Research, School of Medicine, University of Leeds, Leeds LS9 7TF, UK; Leeds Institute for Data Analytics, University of Leeds, Leeds LS9 7TF, USA; Cancer, Ageing and Somatic Mutation, Wellcome Sanger Institute, Hinxton, Cambridgeshire CB101SA, UK; Leeds Institute of Medical Research, School of Medicine, University of Leeds, Leeds LS9 7TF, UK; Leeds Institute for Data Analytics, University of Leeds, Leeds LS9 7TF, USA; Laboratorio Internacional de Investigación sobre el Genoma Humano, Universidad Nacional Autónoma de México, Santiago de Querétaro, México 76230, USA; Cancer, Ageing and Somatic Mutation, Wellcome Sanger Institute, Hinxton, Cambridgeshire CB101SA, UK

## Abstract

A number of genomic regions have been associated with melanoma risk through genome-wide association studies; however, the causal variants underlying the majority of these associations remain unknown. Here, we sequenced either the full locus or the functional regions including exons of 19 melanoma-associated loci in 1959 British melanoma cases and 737 controls. Variant filtering followed by Fisher’s exact test analyses identified 66 variants associated with melanoma risk. Sequential conditional logistic regression identified the distinct haplotypes on which variants reside, and massively parallel reporter assays provided biological insights into how these variants influence gene function. We performed further analyses to link variants to melanoma risk phenotypes and assessed their association with melanoma-specific survival. Our analyses replicate previously known associations in the melanocortin 1 receptor (*MC1R*) and **tyrosinase** (*TYR*) loci, while identifying novel potentially causal variants at the *MTAP/CDKN2A* and *CASP8* loci. These results improve our understanding of the architecture of melanoma risk and outcome.

## Introduction

Cutaneous malignant melanoma (melanoma) is the most aggressive type of skin cancer and originates from melanocytes, the pigment-producing cells of the skin. Ultraviolet radiation exposure is thought to be the main etiological risk factor for melanoma; notably, the number of diagnoses of melanoma has dramatically increased in the recent decades (1). Genetic contributions to this type of cancer have also been identified, mainly through linkage and sequencing studies in the context of high-penetrance disease in melanoma-prone families (2) and by genome-wide association studies (GWASs) in population ascertained cases (3–5).

High-penetrance genetic variants in genes such as cyclin-dependent kinase inhibitor 2A (*CDKN2A*) (6), cyclin-dependent kinase 4 (*CDK4*) (7), telomerase reverse transcriptase (*TERT*) (8), protection of telomeres 1 (*POT1*) (9,10) and other telomere-associated genes are found in approximately half of melanoma-prone families (11), and are known to mediate risk for developing the disease. Common variants that increase the risk of developing melanoma have also been investigated, with the most recent melanoma GWAS meta-analysis including 36 760 cases and 375 188 controls. The authors found 54 genome-wide significant regions (4). However, as these studies are based on genotyping data, the genetic alterations within these regions that increase risk are unknown. The identification of the causal germline genetic lesions that increase cancer predisposition is clinically important, in order to optimize biomarkers for risk stratification, and targeted prevention or treatment (12).

While the GWAS literature contains information on studies with approximately 40 000 cases, inferences on the most associated SNPs are based on imputation. In this study, we identified genetic variants within melanoma-associated haplotypes by sequence analysis of melanoma-associated regions in a British case–control cohort. This strategy has the advantage of giving complete information on regions known to be associated with melanoma to best inform the identification of the most informative and potentially causal SNPs. Our analyses provide insights into critical variants within the *MTAP/CDKN2A* and Caspase 8 (*CASP8*) loci, in addition to confirming already biologically supported variants in the *MC1R* and *TYR* loci. These results refine our knowledge of the genetic architecture of melanoma and contribute to the identification of potential mechanisms of disease development.

## Results

**Figure 1:**
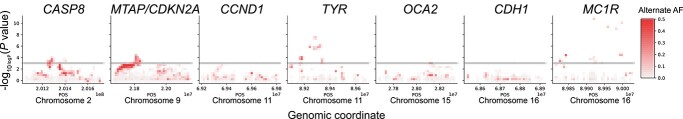
Genomic regions with variants associated with melanoma. Seven genomic association graphs, one per locus, are shown. The *X* axis shows the genomic location and the *Y* axis displays the degree of association (−log_10_(*P*-value)). The horizontal grey line depicts the association threshold (*P* = 0.001). Circle colours represent the allelic frequency of the alternate allele.

We sequenced two full melanoma predisposition loci (methylthioadenosine phosphorylase [*MTAP*]*/CDKN2A*) and *TERT* (see section Methods) as well as the promoters, exons and/or DNAse I hypersensitivity sites (DHSs) associated with genes falling in 17 additional melanoma-associated regions in 1977 melanoma cases and 754 matched controls from the UK population as part of the Leeds Melanoma case–control study (Table 1, section Methods). After variant calling and filtering, we retained 25 329 genetic variants across all regions of interest, of which, only 11 113 had been directly tested in the Landi *et al.* meta-analysis (4). We then removed 18 cases and 17 controls that were not of European descent according to PCA analyses, for a final dataset of 1959 cases and 737 controls. Afterwards, we conducted individual Fisher’s exact tests (see section Methods) of allelic association with melanoma for each of the retained variants and defined 66 single nucleotide polymorphisms (SNPs) as the most informative regarding risk haplotypes at *P* < 0.001 within seven genomic regions (Fig. 1, Supplementary Material, Table S1). In this study, we define a haplotype as the group of all variants within a region whose association with the phenotype depends on the most associated variant in that group (referred to as the lead SNP).

**Table 1:** Loci included in this study

Location (GRCh38)	Genes of interest encoded in the region	Targeted regions
chr1:150754900–151073108	*ARNT, SETDB1*	Exons, promoters and DHSs
chr1:226258651–226499883	*PARP1*	Exons, promoters and DHSs
chr2:201047085–201724509	*CASP8, ALS2CR12, CFLAR, TRAK2*	Exons, promoters and DHSs
chr4:24692289–25055958	*SOD3, CCDC149*	Exons and promoters
chr5:1142197–1447788	*TERT, CLPTM1L*	Full locus
chr5:33840139–34064027	*SLC45A2*	Exons, promoters and DHSs
chr6:83948233–84292890	*MRAP2, KIAA1009*	Exons and promoters
chr9:21665209–22151817	*MTAP, CDKN2A, CDKN2B, CDKN2B-AS1/ANRIL*	Full locus
chr11:69210414–69803433	*CCND1, MYEOV, ORAOV1, FGF19*	Exons, promoters and DHSs
chr11:89097917–89675699	*TYR, NOX4*	Exons, promoters and DHSs
chr11:108136364–108468324	*ATM*	Exons, promoters and DHSs
chr15:27670078–28337214	*OCA2, HERC2*	Exons and promoters
chr16:53363904–54384452	*FTO, IRX3, RBL2*	Exons, promoters (*FTO, IRX3, RBL2*) and DHSs (*FTO* and *IRX3*)
chr16:68528729–68923897	*CDH1, CDH3*	Exons and promoters
chr16:89806464–90031424	*MC1R*	Exons and promoters
chr17:80154433–80508392	*RNF213*	Exons and promoters
chr20:33709976–34374270	*ASIP, RALY, EIF2S2, CHMP4B*	Exons, promoters and DHSs
chr21:41194545–41512249	*MX2, FAM3B*	Exons, promoters and DHSs
chr22:37870877–38328731	*PLA2G6, SOX10, PICK1, SLC16A8*	Exons, promoters and DHSs

The location (GRCh38), genes of interest and the targeted regions for each locus are indicated.

For all associated variants in each region, we performed stepwise conditional logistic regression to detect the distinct haplotypes on which these variants are located. In order to identify potential biological mechanisms through which these variants may act, we also conducted association trend tests with a number of melanoma-linked phenotypes in the UKBiobank and ENGAGE cohorts (section Methods). For candidate variants in the *MTAP/CDKN2A* and *CASP8* regions, we also integrated previously published data from a massively parallel reporter assay (MPRA) designed to identify risk-associated variants with allelic *cis*-regulatory activity (13). Finally, we also evaluated melanoma-specific survival (MSS) in carriers and non-carriers of melanoma-associated lead variants in the Leeds Melanoma Cohort (LMC, Methods). In the following sections, we discuss the *MC1R* and *TYR* regions in detail as proof of concept and *MTAP/CDKN2A* and *CASP8* where we identified novel potential candidate variants. Results for the other regions where associated variants were identified can be found in the Supplementary Note.

### Proof of concept: fine-mapping of the melanocortin 1 receptor and tyrosinase loci identifies established risk variants

**Figure 2:**
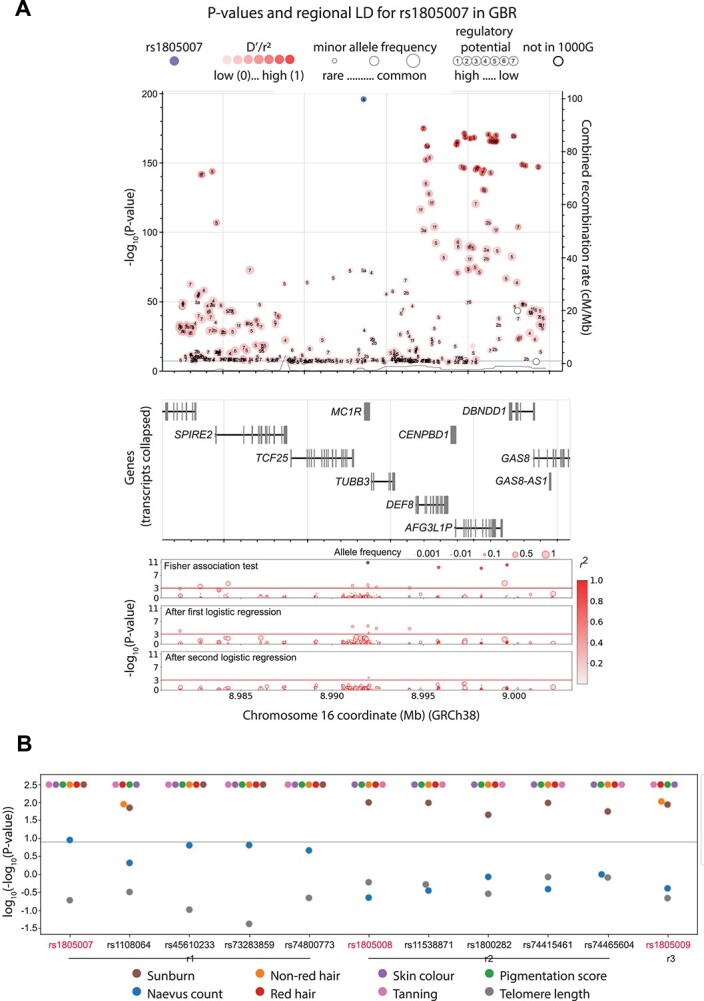
Association of variants in the *MC1R* region with melanoma, melanoma-linked phenotypes and survival. (**A**) The top panel depicts the −log_10_(*P* value) for association with melanoma of those variants most strongly associated (*P* value < 1 × 10^−7^) from the most recent melanoma meta-analysis (4). Numbers inside the circles display the regulomeDB (49) rank, the circle size represents the minor allele frequency. The variant in purple is the most associated in the meta-analysis (rs1805007), the red shade depicts the linkage disequilibrium (*r*^2^) value with respect to it. The horizontal blue line indicates *P* = 5 × 10^−8^. The overlay *Y* axis depicts the combined recombination rate from HapMap (50). The middle panel depicts the genes in the genomic region. These panels were interactively plotted by LD-assoc (https://ldlink.nci.nih.gov/) (51). The lower panel shows the results of the association tests and the logistic regression in the melanoma case–control cohort in this study. The colour of the circle represents the linkage disequilibrium (*r^2^*) value with respect to the most associated variant, and the circle size represents the allelic frequency of the minor allele. (**B**) *P*-value of association tests of all melanoma-associated variants with distinct melanoma-related phenotypes. r1, r2 and r3 represent distinct haplotypes, *P*-values were truncated at log_10_(−log_10_ > 2.488). The gray line represents *P* value = 1 × 10^−8^.

The locus most highly associated with melanoma in our dataset was *MC1R* (Fig. 1), which reflects previous reports (5). As part of our experiment, we targeted all exons and the promoter of *MC1R*. Subsequent association tests revealed 10 variants to be associated with melanoma (Fisher’s exact tests *P* < 0.001, Fig. 2A, Supplementary Material, Table S1), identifying these variants as the most informative for melanoma risk in this dataset. Stepwise conditional logistic regression was then deployed to identify three independent haplotypes in this region, with the lead SNPs for these three independent signals corresponding to the known *MC1R* melanoma-predisposing alleles p.Arg151Cys (rs1805007), p.Arg160Trp (rs1805008) and p.Asp294His (rs1805009) (Fig. 2A). The allele frequencies (AFs) and odds ratios (ORs) calculated from this dataset closely reflect those reported in the literature (Fig. 2A, Supplementary Material, Table S1).

Next, we assessed the association of these SNPs with known melanoma-linked phenotypes using the UKBiobank and ENGAGE datasets, in order to search for clues to the biological mechanisms through which they may be acting to increase melanoma risk. The minor alleles of the three leading SNPs in *MC1R* haplotypes (rs1805007, rs1805008 and rs1805009), as well as the other eight associated SNPs, are all associated with hair colour: red hair against all others, number of incidents of childhood sunburn, skin pigmentation and tanning ability at genome-wide significance (Fig. 2B, Supplementary Material, Table S2). These are all established associations given the biological role that *MC1R* plays in melanin production and the role of melanin in promoting the tanning response to protect DNA from damage (14). The lead SNP rs1805007 was also associated with naevus count; this was observed to a lesser degree with the other SNPs in its haplotype. None of the tests for association with survival was significant (*P* adjusted for multiple testing <0.05) (Supplementary Material, Table S3).

**Figure 3:**
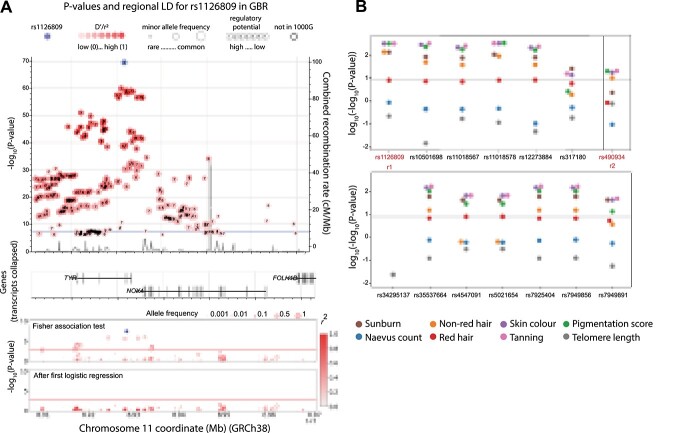
Association of variants in the *TYR* region with melanoma and melanoma-linked phenotypes. (**A**) The top panel depicts, in the *Y* axis, the −log_10_(*P* value) for association with melanoma of those variants most strongly associated (*P* value < 1 × 10^−7^) from the most recent melanoma meta-analysis (4) as well as the combined recombination rate from HapMap (50). Numbers inside the circles hold regulatory information (if any) from RegulomeDB (49), the circle size represents the minor allele frequency. The variant in purple is the most associated in the meta-analysis (rs1126809), the red shade depicts the linkage disequilibrium (*r*^2^) value with respect to it. The horizontal blue line indicates *P* = 5 × 10^−8^. The genes in the genomic region are depicted in the middle panel. These panels were interactively plotted by LDassoc (https://ldlink.nci.nih.gov/) (51). The results of the association tests and logistic regression in the melanoma case–control cohort in this study are shown in the lower panel. The colour of the circle represents the linkage disequilibrium (*r*^2^) value with respect to the most associated variant, and the circle size represents the allelic frequency of the minor allele. (**B**) *P*-value of association tests of all melanoma-associated variants with distinct melanoma-related phenotypes. r1 and r2 represent distinct haplotypes, *P*-values were truncated at log_10_(−log_10_ > 2.488). The gray line represents *P* value = 1 × 10^−8^.

For the tyrosinase locus (*TYR*), we sequenced the promoter, all exons and nearby DHSs from the *TYR* and *NOX4* genes (see section Methods). Overall, the association test found 14 variants associated with melanoma (Fisher’s exact tests *P* < 0.001), all of them in the same haplotype led by rs1126809 (Fig. 3A, Supplementary Material, Table S1). This variant codes for p.Arg402Gln, a known melanoma predisposition allele (15). After the first logistic regression, the next most highly associated variant was rs490934 (association *P* < 0.05), which falls in an intron in *NOX4*. As expected, all assessable variants in the first haplotype are associated with number of incidents of childhood sunburn, ease of skin tanning and skin pigmentation, while rs490934 was associated with the same phenotypes with the exception of childhood sunburn (Fig. 3A). No significant associations with patient survival were found (*P* adjusted for multiple testing <0.05) (Supplementary Material, Table S3). The lead SNPs we identified in *MC1R* and *TYR* are the same ones identified by Landi *et al.* in their 2020 meta-analysis (4). These results indicate that we are able to identify melanoma-associated germline alleles and patient phenotypes related to outcomes of biological mechanisms through which such alleles may act.

### MTAP/CDKN2A region

**Figure 4:**
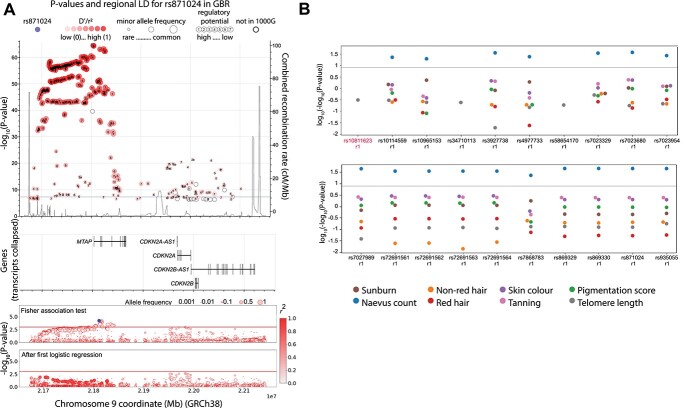
Association of variants in the *MTAP/CDKN2A* region with melanoma melanoma-linked phenotypes and survival. (**A**) The top panel shows the combined recombination rate from HapMap and the −log_10_(*P*-value) of association with melanoma for those variants most strongly associated (*P* value 1 × 10^−7^) from the most recent melanoma meta-analysis (4) as well as the combined recombination rate from HapMap (50). Numbers inside the circles hold regulatory information (if any) from RegulomeDB (49), the circle size represents the minor allele frequency. The purple dot is the most associated in the meta-analysis (rs871024), the red shade depicts the linkage disequilibrium (*r*^2^) value with respect to it. The horizontal blue line indicates *P* = 5 × 10^−8^. The genes in the genomic region are depicted in the middle panel. These panels were interactively plotted by LDassoc (https://ldlink.nci.nih.gov/) (51). The results of the association tests and logistic regression in the melanoma cohort in this study are shown in the lower panel. The colour of the circle represents the linkage disequilibrium (*r*^2^) value with respect to the most associated variant, and the circle size represents the allelic frequency of the minor allele. (**B**) *P*-value of association tests of all melanoma-related variants with distinct melanoma-linked phenotypes. r1 and r2 represent distinct haplotypes. The gray line represents *P* value = 1 × 10^−8^.

The *MTAP/CDKN2A* locus encompasses two functional tumour suppressor genes, *CDKN2A* and *CDKN2B*, that are known to play a role in familial and sporadic melanoma risk, as well as a number of other genes with potential involvement in this disease. For this region, we targeted the full genomic space between GRCh38 chr9:21665209–22151817, which included *MTAP*, *CDKN2A*, *CDKN2B* and *CDKN2B-AS1/ANRIL* as genes of interest (Table 1, Methods). Overall, our association tests identified 22 SNPs associated with melanoma (Fisher’s exact test *P* < 0.001, Supplementary Material, Table S1, Fig. 4A). Of these, all but one, fell into one haplotype, with lead SNP rs10811623, an intronic variant in the *MTAP* gene that lies within a large promoter-flanking regulatory region. Only SNP rs35302371 fell into a different haplotype (association *P*-value after first logistic regression <0.0023), which lies in an intron within *CDKN2A*. All the SNPs in both haplotypes that could be evaluated for their association to melanoma-linked phenotypes showed an association with naevus count (Supplementary Material, Table S2, Fig. 4B). MPRAs performed on ten of these SNPs show regulatory potential for rs80138396 (with a significant allelic difference) (Supplementary Material, Table S4). This SNP is in moderate (*r*^2^ = 0.58, GBR population) linkage disequilibrium (LD) with our lead SNP (rs10811623) and complete LD (*r*^2^ = 1, GBR population) with rs871024, which is the one identified as the lead by Landi *et al*. (4). In our analysis, rs871024 is also found to be associated with melanoma (*P*-value 6.2 × 10^−4^) with a similar OR (1.23, compared to 1.18 in Landi *et al.* (4)). According to the LD-pop tool (16), the LD in the British population between rs10811623 and rs871024 (*r*^2^) is 0.59 in the 1000 Genomes Project, indicating they are moderately correlated. This suggests that both rs10811623, as the most highly associated SNP in this study, and rs80138396 could potentially encode functional alleles in this region. The lead SNP for the second haplotype, rs35302371, has not been previously identified as associated with melanoma to the best of our knowledge.

An important issue in melanoma genetics is the classification of novel variants in known risk genes. Fifteen *CDKN2A* coding variants were identified in this study (Supplementary Material, Table S5), including one novel, heterozygous variant in a case (p.Trp160Arg), predicted deleterious by the SIFT algorithm and with unknown significance by PolyPhen-2 (17,18), a rare frameshift variant in a case (rs779306249, p.Glu33GlyfsTer30) and an inframe variant in a case (rs779983400, p.Glu113del). The only coding variant moderately associated with melanoma was rs3731249 (p.Ala148Thr, OR: 1.5, *P*-value = 0.035), which has been described as a risk variant previously in other populations (3,19).

### CASP8 region

**Figure 5:**
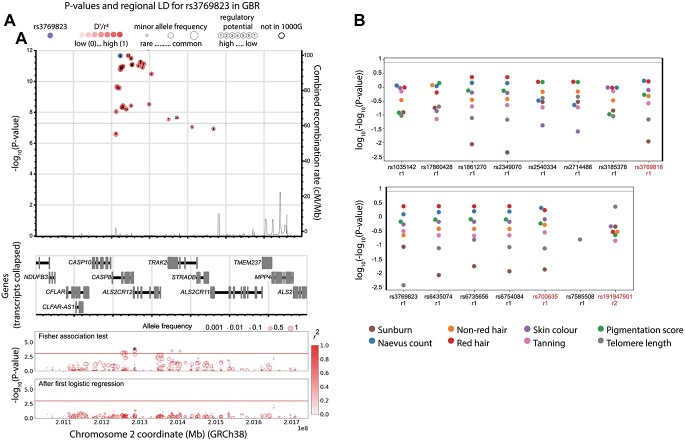
Association of variants in the *CASP8* region with melanoma melanoma-linked phenotypes and survival. (**A**) The top panel shows the combined recombination rate from HapMap and the −log_10_(*P* value) of association with melanoma for those variants most strongly associated (*P* value 1 × 10^−7^) from the most recent melanoma meta-analysis (4) as well as the combined recombination rate from HapMap (50). Numbers inside the circles hold regulatory information (if any) from RegulomeDB (49), the circle size represents the minor allele frequency. The purple dot corresponds to rs3769823, the red shade depicts the linkage disequilibrium (*r*^2^) value with respect to it. The horizontal blue line indicates *P* = 5 × 10^−8^. The genes in the genomic region are depicted in the middle panel. These panels were interactively plotted by LDassoc (https://ldlink.nci.nih.gov/) (51). The results of the association tests and logistic regression in the melanoma cohort in this study are shown in the lower panel. The colour of the circle represents the linkage disequilibrium (*r*^2^) value with respect to the most associated variant, and the circle size represents the allelic frequency of the minor allele. (**B**) *P*-value of association tests of all melanoma-associated variants with distinct melanoma-related phenotypes. r1 and r2 represent distinct haplotypes. The gray line represents *P* value = 1 × 10^−8^.

For this region, we targeted the promoter, exons and associated DHSs of all genes of interest overlapping GRCh38 chr2:201047085–201724509, which include *CASP8*, *ALS2CR12* (Also known as *FLACC1*), *CFLAR* and *TRAK2*. Overall, our association tests identified 16 SNPs associated with melanoma (Fisher’s exact tests *P* < 0.001, Fig. 5A, Supplementary Material, Table S1, Methods) in one haplotype with lead SNPs rs3769818 and rs700635, and, after the first logistic regression, the next most highly associated variant was SNP rs191947901 (*P* < 0.004). No associations were found for any of these SNPs with melanoma-linked phenotypes (Fig. 5B), nor with survival (*P* adjusted for multiple testing >0.05) (Supplementary Material, Table S3). Landi et al. (4) identified rs10931936 as the lead SNP in this region, which was not captured in our data as our experiment preceded their publication. However, according to LD-pop, these are all in complete LD in the British population in the 1000 Genomes Project (*r^2^* = 1). A missense variant in *CASP8*, rs3769823 coding for p.Lys14Arg and which lies in a vitamin D binding site (20), was also found in this haplotype. This variant, though moderately correlated with the lead SNPs (*r*^2^ = 0.6917), has been suggested to explain the signal for basal cell carcinoma risk at this locus (19). MPRAs also showed that the reference allele (A) is functional and associated with *cis*-regulatory activity as an activator (Supplementary Material, Table S4). Therefore, rs3769823 is a plausible candidate for being the causal SNP in this region, both because it alters the protein sequence of caspase 8 and because it appears to be cis-regulatory.

### Other regions

Variants highly associated with melanoma were also found in three other regions: oculocutaneous albinism 2 (*OCA2*, chr15:27670078–28337214), E-cadherin (*CDH1*, chr16:68528729–68923897) and cyclin D1 (*CCND1*, chr11:69210414–69803433) (Supplementary Material, Figs S1–S3). Two, one and one associated variants were found, respectively (Supplementary Note).

## Discussion

Predicting an individual’s risk of disease provides an opportunity to preempt surveillance, to counsel patients about behaviours that may mitigate their likelihood of developing a condition, and also reveals fundamental insights into disease biology. In this study, we perform the largest melanoma resequencing project ever conducted to our knowledge, exploring the allelic landscape of melanoma loci by analyzing nearly 2700 individuals. Our analysis identifies established melanoma risk variants such as disruptive coding alleles of *MC1R* and *TYR*. Importantly, we were also able to extend our analysis of germline alleles in melanoma patients to identify candidate functional variants at loci including *MTAP/CDKN2A,* where risk alleles were associated with phenotypes such as naevus count, and *CASP8*.

In performing the studies outlined in our paper, we aimed to illuminate biological insights into how melanoma risk loci contribute to disease development. One notable observation we made was that the lead variant at the *CASP8* locus (rs3769818) was associated with melanoma (G allele) but not with melanoma-linked phenotypes, such as tanning or naevus count. By analysis of MPRA data, the linked variant rs3769823, also identified in this study, was found to be associated with altered *cis*-regulatory activity and to fall into a canonical vitamin D binding site. More specifically, the rs3769823-A allele, which is linked to rs3769818-A, appears to be associated with *cis*-regulatory activity as an activator. Therefore, this variant may activate the expression of *CASP8*, a gene which plays a key role in programmed cell death mediating processes such as apoptosis, necroptosis and pyroptosis (21). However, it is the alternate allele (G) that shows an association with higher *CASP8* levels in both a previously published eQTL analysis in primary melanocyte cultures (22) and a range of tissues in the GTex database, as well as lower *ALS2CR12* levels in thyroid tissue (23). *CASP8* has also been found to be overexpressed in a range of cancers including prostate and renal cancer where it is associated with a poor outcome. A potential explanation for this observation could be the role of CASP8 in increasing NF-κB levels, an event we (24) and others (25,26) have previously linked to enhanced melanoma cell growth. Thus, individuals who carry rs3769823-G may be predisposed to melanoma development via a role for this variant in influencing a switch from pro-apoptotic to anti-apoptotic functions of NF-κB. The potential role of vitamin D signalling in this context requires further exploration with an extensive literature linking low vitamin D levels to both risk of melanoma development and poor melanoma-associated outcomes (27).

In the *MTAP*/*CDKN2A* region, we identified two potentially causal SNPs by integrating results from this study, that of previously generated MPRA tests and the literature. The first one, rs80138396, is completely correlated with the lead SNP identified by Landi *et al.* (*r*^2^ = 1) and shows regulatory potential by MPRA assays, and thus may explain that association signal. The second SNP, rs10811623, appears as the most strongly associated with melanoma in this sequencing study and falls within a large promoter-flanking regulatory region. SNPs within the first haplotype also show association with nevus count, which is of interest given that familial atypical multiple mole melanoma syndrome (FAMMM), a condition characterised by multiple melanocytic nevi, is associated with germline variants in the nearby *CDKN2A* gene.

Thus, in this study, we provide a detailed exploration of melanoma risk loci and biological insights into the processes that drive this malignancy. These data provide a foundation for further functional studies such as CRISPR genome-editing of risk loci or other *in vivo* or *in vitro*-based experiments that, in the near future, should deepen our knowledge of the influence of genetic variants on melanoma development.

## Materials and Methods

### Ascertainment of cases and controls

Ethical approval for the Leeds Melanoma Cohort Study was granted by the North East—York Research Ethics Committee (MREC 1/3/57), the Patient Information Advisory Group (PIAG 3–09(d)/2003) and further approved by the Wellcome Sanger Institute Human Materials and Data Management Committee. Patients have been part of previous GWAS melanoma projects (3–5), and the inclusion criteria for patients have been previously published (28). Briefly, population-ascertained incident melanoma cases in a geographically defined area of Yorkshire and the northern region of the UK were recruited to a case–control study to form the Leeds Case Control cohort; and from these, germline DNA was obtained from 1977 cases and 488 controls along with extensive patient clinical data. Two hundred and sixty-six samples from control subjects were also obtained through the Wellcome Trust Case Control Consortium (WTCCC) (29) for a total number of 754 controls. We then further filtered our dataset by ethnicity using both self-reporting and principal component analysis to retain 1959 cases and 737 controls, ensuring that all individuals in this study are confirmed to be of European origin (Supplementary Material, Fig. S4, Supplementary Note).

### Selection of candidate regions

Nineteen regions previously reported as associated regions, from which 15 were genome-wide significant loci from a melanoma GWAS meta-analysis (5) and four that were identified as candidate melanoma susceptibility regions by a preliminary version of the same GWAS, were selected for inclusion in a custom designed sequencing panel (Table 1). All genes falling within the limits of linkage disequilibrium around the melanoma GWAS-associated region were included in the panel. Exons, promoters and DNAse I hypersensitivity sites associated with these genes were included as follows:

- Exons. For every gene of interest in each associated region, all exons belonging to it, regardless of the transcript biotype (e.g*.* protein-coding or others) as annotated in Ensembl (30) release 69 were included. 100 bp of intron either side of each exon was included to ensure the appropriate capture of potential splice site alterations.
- Promoters. For each gene, the transcription start site (TSS) as annotated in Ensembl release 69 was noted. Additional TSSs per gene were manually curated to include other possible alternative TSSs associated with non-canonical transcripts in the gene. From all these, 5000 bp upstream to the TSS were included in the design (Supplementary Material, Table S6).
- DNAse I hypersensitivity sites (DHS). For each included gene, a window of 100 kb of sequence around the gene was searched for DHSs annotated in melanoma cells profiled by ENCODE (31). The cell lines that were considered in this search were Colo829, Mel2183, Melano and RPMI7951. No thresholding was applied to this selection, and the union of all annotated regions in all cell lines was included (Supplementary Material, Table S7).
- Full loci. Two full loci containing the limits of linkage disequilibrium around the GWAS signal were included in this design. The *MTAP/CDKN2A* region was defined from GRCh38 chr9:21665209–22151817, and the *TERT* region was defined from chr5:1142197–1447788.

In total, the assessed genomic space amounted to 2.061 Mb. Target region coordinates were submitted through the SureDesign platform to Agilent Technologies for their manufacture with a tiling density of 2×, ‘moderately stringent’ masking and ‘maximize performance’ boosting. Information associated with this design is included in Supplementary Material, Table S8.

### Targeted DNA sequencing

DNA was extracted from blood from consenting participants and processed as previously reported in Newton-Bishop *et al*. (32). Sequencing was done in two batches using the Illumina HiSeq platform. Reads were aligned to the GRCh38 reference genome using the BWA-MEM algorithm (33), achieving comparable coverage in cases and controls in all regions. Duplicate marking was done using biobambam streaming markduplicates (34).

### Variant calling and quality control

For each aligned file, the GATK v4.1.0.0 HaplotypeCaller was run to create a genomic variant call format (gVCF) file. For joint calling, the individual target regions were combined into 103 batches. Each batch (20 target regions) was supplied to the GATK GenomicsDBImport tool along with all the gvcf called in the previous step. The resulting genomicsDB was then supplied to the GATK GenotypeGVCFs tool, creating a single vcf file with genotypes for all samples for all target regions in the batch. These steps were performed for all 103 batches. The vcf files for all batches were combined together using bcftools concat v1.9 (35) to create a final single vcf file containing 40 936 variants. Samples were separated into sequencing batches and phenotype cohort (cases and controls). The following filters were applied for variants in each batch, and each cohort (four groups in total):

- A *P*-value from a Hardy–Weinberg Equilibrium test (36) was calculated for each variant site using PLINK v1.90b3v (www.cog-genomics.org/plink/1.9/) (37) separately in both control batches. All variants with *P* < 0.001 were excluded.
- Average GQ values were calculated (38). All variants with an average GQ < 30 were filtered out. Sites with more than 0.05 missing genotypes on the basis of their proportion were also filtered out.

Batches were joined keeping only variants that were common among them after these filters. Then further filters were applied:

- All variants overlapping low-complexity regions (LCRs) (39) were filtered out.
- For each site, the rate of genotype missingness was calculated and a Fisher’s exact test (37) was performed between cases and controls; any site with *P* < 0.05 was excluded.
- The probability of the called samples exhibiting excess heterozygosity with respect to the null hypothesis that the samples are unrelated was estimated using the GATK v4.1.1.0-ExcessHet (40). All variants had an ExcessHet value below 54.96, which corresponds to a *P*-value of 3.4 × 10^−06^.
- The GATK v4.1.1.0 Variant Quality Score Recalibration (VQSR) (40) was applied following their Best Practices for exome sequencing (41). We retained variants in the 99.9% tranche sensitivity threshold for both SNVs and indels. Tranche plots for the SNVs can be seen in Supplementary Material, Figure S5.
- All variants that overlapped alterations found in the gnomAD database version 3.0 (42), but that were annotated as not passed in this database, were filtered out. This filter was included to eliminate potential systematic artefacts that can be detected by gnomAD given its scale.
- Finally, the allele frequency (AF) of each variant site in controls was compared to that reported for the non-Finnish European population (NFE) in the gnomAD dataset (42). The difference in AF between both populations was converted into z-scores using the following formula: *z* = (*X* − *μ*)/*σ*, where *x* is a single data value, *μ* is the population mean and *σ* is the population standard deviation. Any site with a *z*-score ≤−9 or ≥9 was filtered out (Supplementary Material, Fig. S6).

In the end, a set of 25 329 high-quality variants were selected for further analyses.

### Variant association analyses

Individual Fisher’s exact tests of allelic association with the phenotype were conducted for each variant. No adjustments were made for multiple comparisons: as these regions were selected on the basis of a previously established association, a *P* < 0.001 was considered to indicate an informative variant leading the association of an identifiable haplotype. In order to detect clusters of variants of interest, a stepwise logistic regression controlling for the most strongly associated variant in each region was performed for those regions with more than one variant passing the defined threshold. For this cluster selection, a threshold *P*-value was considered to indicate association with the variants selected to condition the logistic regression model. This was set to *P*-value < 0.1. PLINK v1.90b3v (www.cog-genomics.org/plink/1.9) (37) was used for these statistical analyses. The aligned read sequences of all variants passing the threshold of association were manually inspected using Samtools (43) tview to detect potential mapping errors, resulting in 73 variants that passed these filters (These include the next most highly associated variant and any additional variants passing the predetermined threshold after applying logistic regression.) BCFtools (43) and VCFtools (44) were used to facilitate the data handling. In this study, we use the term ‘haplotype’ to indicate the variants within a region whose association to the phenotype depends on the most associated variant (referred to as the lead SNP).

### Association of each of the variants identified above with melanoma risk phenotypes in a larger cohort

To investigate their contribution to the known melanoma risk phenotypes, candidate variants were tested for their association with various pigmentation phenotypes: skin pigmentation, childhood sunburn occasions, hair colour: red against all others, hair colour: blond, light brown, dark brown, black, ease of skin tanning and combined pigmentation score (first principal component of a PCA of all of these), using the UK Biobank data as previously described (4) for hair colour, and which involves treating traits with more than two values as ordinal variables. In addition, the association of the candidate variants to telomere length was also assessed using GWAS data from a meta-analysis by the ENGAGE consortium (45). The association of the candidate variants to naevus count was done using the naevus count data included in the Landi *et al*. melanoma meta-analysis paper (4).

### Genetic association with melanoma outcomes

Candidate SNPs tagging the haplotypes most strongly associated with melanoma risk were tested for association, in the same case–control cohort, with melanoma-specific survival (MSS), age, sex, tumour thickness, ulceration and mitotic rate. MSS was assessed by examination of clinical records and national death records; when deaths were recorded, a clinical assessment of the causes of death was made to assess if the death could be attributed by melanoma. Deceased patients were censored at time of death; live patients were censored at the cut-off date for this analysis or the time last known to be alive if contact had been lost with the participant. We found no association with any of these variables. The median follow-up time within the LMC for MSS information was approximately 8 years at the time of this analysis; information was available on all participants (46). Univariable Cox proportional hazards regression was applied and Kaplan–Meier survival curves were plotted in STATA v14 (47) comparing two groups: carriers vs non-carriers of the risk allele.

### Functional testing of variants in MTAP/CDKN2A and CASP8 via massively parallel reporter assays

Data generated previously as reported by Choi and colleagues (13) were paired to the candidate risk haplotypes for both regions. The overlapping matches associated or in linkage disequilibrium with the haplotypes here reported were assessed in their allelic transcriptional function with the MPRA approach. In brief, variants that are in LD (*r*^2^ > 0.4) with GWAS leads from the melanoma meta-analysis conducted by Law and colleagues (5) and secondary leads identified through fine-mapping were selected. The potential as an enhancer or promoter element was tested using a 145-bp genomic sequence encompassing each variant site and was investigated in luciferase constructs. Each allele of each variant, designed in both forward and reverse directions, was linked to 10 unique barcode sequences. The surrounding 20 bp of each variant was scrambled to be tested as a null. The transcription of barcodes of each element transfected into a melanoma cell line (UACC903, to represent melanoma-specific *trans*-acting factors) as well as the cell line HEK293FT (to represent non-cell-type-specific activity in a highly transfectable cell line) was measured by sequencing. Variants with a significant difference between two alleles comparing the ratio of RNA TPM/DNA TPM (tags per million, e.g. normalized tag count) are reported as those with allelic transcriptional activity (FDR < 0.01; two-sided Wald test with robust sandwich type variance estimate; multiple comparisons adjusted using Benjamini and Hochberg method) (48).

## Supplementary Material

Supplementary_Figure_1_ddac074

Supplementary_Figure_2_ddac074

Supplementary_Figure_3_ddac074

Supplementary_Figure_4_ddac074

Supplementary_Figure_5_ddac074

Supplementary_Figure_6_ddac074

Supplementary_Table_1_ddac074

Supplementary_Table_2_ddac074

Supplementary_Table_3_ddac074

Supplementary_Table_4_ddac074

Supplementary_Table_5_ddac074

Supplementary_Table_6_ddac074

Supplementary_Table_7_ddac074

Supplementary_Table_8_ddac074

Supplemental_data_ddac074

## Data Availability

Sequence data for all cases and controls in this study have been deposited at the European Genome-phenome Archive and can be found under accession number EGAD00001007520. All code used in this study can be found in https://github.com/citosina/melanoma_reseq. MPRA data are available in the NCBI Gene Expression Omnibus as a SuperSeries under the accession number GSE129250.
