# Supplementary material for: Defining novel causal SNPs and linked phenotypes at melanoma-associated loci
Source: Hum Mol Genet. 2022 Mar 31;31(17):2845–56. doi: 10.1093/hmg/ddac074 (PMC9433725; doi:10.1093/hmg/ddac074)
Supplement: Supplemental_data_ddac074 [file supplemental_data_ddac074.doc]

**Supplementary Figure Legends**

**Supplementary Figure 1. Association of variants in the *OCA2* region with melanoma melanoma-linked phenotypes. a)** Regional association *P*-values for those identified melanoma susceptibility variants most strongly associated (*P*-value 1x10^-7^) from the most recent melanoma meta-analysis(4) is depicted in the top panel as well as the combined recombination rate from HapMap in the shared Y axis. The numbers contained in the circles represent RegulomeDB (49) [(Boyle et al., 2012)](https://www.zotero.org/google-docs/?p2QgV7) regulatory classification, and the circle size reflects minor allele frequency. The purple circle corresponds to rs129123832, the top SNP in the region reported in the latest meta-analysis [(Landi et al., 2020)](https://www.zotero.org/google-docs/?YuAark). The red shade depicts the linkage disequilibrium (r^2^) value with respect to it. The horizontal blue line indicates *P* =5× 10^−8^. The genes in the genomic region are depicted in the middle panel. These panels were interactively plotted by Ldassoc (<https://ldlink.nci.nih.gov/>) (51). The results of the association tests and logistic regression in the melanoma cohort in this study are shown in the lower panel. The colour of the circle represents the linkage disequilibrium (r^2^) value with respect to the most associated variant, and the circle size represents the allelic frequency of the minor allele. **B)** P-value of association tests of all melanoma-associated variants with distinct melanoma-related phenotypes. r1 and r2 represent distinct haplotypes, *P*-values were truncated at log_10_(-log_10_ > 2.488). The gray line represents *P* value =1x10^-8^.

**Supplementary Figure 2. Association of variants in the *CDH1* region with melanoma melanoma-linked phenotypes and survival. a)** Regional association *P*-values for those identified melanoma susceptibility variants most strongly associated (*P*-value 1x10^-7^) from the most recent melanoma meta-analysis(4) is depicted in the top panel as well as the combined recombination rate from HapMap in the shared Y axis. The numbers contained in the circles represent RegulomeDB (49) regulatory classification, and the circle size reflects minor allele frequency. The purple circle corresponds to rs4420522, the top SNP in the region reported in the latest meta-analysis. The red shade depicts the linkage disequilibrium (*r*^2^) value with respect to it. The horizontal blue line indicates *P* =5× 10^−8^. The genes in the genomic region are depicted in the middle panel. These panels were interactively plotted by LDassoc (<https://ldlink.nci.nih.gov/>) (51). The results of the association tests and logistic regression in the melanoma cohort in this study are shown in the lower panel. The colour of the circle represents the linkage disequilibrium (*r*^2^) value with respect to the most associated variant, and the circle size represents the allelic frequency of the minor allele. **b)** *P*-value of association tests of all melanoma-related variants with distinct melanoma-linked phenotypes. r1 and r2 represent distinct haplotypes. The gray line represents *P* value =1x10^-8^.

**Supplementary Figure 3. Association of variants in the *CCND1* region with melanoma melanoma-linked phenotypes. a)** Regional association *P*-values for those identified melanoma susceptibility variants most strongly associated (*P*-value 1x10^-7^) from the most recent melanoma meta-analysis (4) is depicted in the top panel as well as the combined recombination rate from HapMap in the shared Y axis. The numbers contained in the circles represent RegulomeDB (49) regulatory classification, and the circle size reflects minor allele frequency. The purple circle corresponds to rs4354713, the red shade depicts the linkage disequilibrium (r^2^) value with respect to it. The horizontal blue line indicates *P* =5× 10^−8^. The genes in the genomic region are depicted in the middle panel. These panels were interactively plotted by LDassoc (<https://ldlink.nci.nih.gov/>) (51). The results of the association tests and logistic regression in the melanoma cohort in this study are shown in the lower panel. The colour of the circle represents the linkage disequilibrium (*r*^2^) value with respect to the most associated variant, and the circle size represents the allelic frequency of the minor allele. **b)** *P*-value of association tests of all melanoma-associated variants with distinct melanoma-related phenotypes. r1 and r2 represent distinct haplotypes. The gray line represents *P* value =1x10^-8^.

**Supplementary Figure 4. Principal component analysis plot for cases and controls included in this study.** Samples are plotted alongside HapMap3 samples. Gray points indicate samples in this cohort, red points are Tuscan Italians (TSI), magenta points are South Asians and Mexicans (GIH/MXL), orange points represent East Asians (CHB/CHD/JPT), green points represent Africans (ASW/LWK/MKK/YRI) and black points represent European samples (CEU) (these are all underneath the gray points). The vertical dashed line was the cut-off used for excluding non-European samples.

**Supplementary Figure 5. Tranche plots for Variant Quality Score Recalibration (VQSR).** The threshold selected for this study was 99.9.

**Supplementary Figure 6. Comparison of allelic frequencies of variants identified in this study and variants annotated in GnomAD.** Variants that passed this filter are depicted. The allelle frequency in this study is given on the X axis whiles the allele frequency in GnomAD is given on the Y axis.

**Supplementary Table Legends**

**Supplementary Table 1. List of variants most strongly associated with melanoma in this study.** The column ‘INFO’ includes information about the variant consequences, including gene symbol and Ensembl transcript ID (Ensembl release 99) (30). The ‘P-value’ column has the Fisher’s test association value, ‘P value 1st’ and ‘P value 2nd’ refer to the P-values after the first and second logistic regression analyses respectively, controlling for the most associated variant. AF: Allele frequency, OR: Odds ratio. The ‘rx’ nomenclature in the ‘Region’ column refers to variants in the same haplotype (‘r1’ is the highest associated haplotype, ‘r2’ the second highest, etc.).

**Supplementary Table 2. Association between associated SNPs and melanoma-linked traits. ‘**REF’ refers to the reference allele, ‘ALT’ is the alternative allele. OR: Odds ratio, SE: Standard error, P_P-value, BETA: Beta coefficient from linear regression. Sunburn: sunburn occasions during childhood, Skin: skin pigmentation, tanning: Ease of tanning, pigscore: pigmentation score from PCA, TEL: Telomere length, NAEV: naevus count.

**Supplementary Table 3. Survival analyses results from Cox-proportional hazards modeling of melanoma-specific survival.** Results are shown for the lead SNPs in each region, as well as analysis of melanoma severity phenotypes among cases (stage and Breslow thickness at diagnosis).

**Supplementary Table 4. MPRA results for selected variants in the *MTAP/CDKN2A* and *CASP8* regions.** 'Function' refers to the inferred function of a SNP, activator means that the alternative allele is significantly associated to higher mRNA expression of the target gene. 'UACC903/HEK293' refers to analyses considering both cells together, 'UACC903 Only' refers to these cells considered alone. FDR: False discovery rate.

**Supplementary Table 5. Coding variants in *CDKN2A* found in this study.** Different lines per variant are shown if more than one transcript is affected by the variant.

**Supplementary Table 6. Transcription start sites annotated for each gene of interest.** Coordinates are in GRCh37, as this annotation was done with Ensembl release 69.

**Supplementary Table 7.** **Coordinates (GRCh37) of DNAse I Hypersensitivity sites captured per targeted gene.**

**Supplementary Table 8. Design parameters for the capture probes used in this study.** This table includes all relevant parameters as reported by Agilent Technologies. The coordinates for all targeted regions can be found after the design, target and probe summary information.

**Supplementary note**

Variants associated with melanoma were also found in these regions:

*Oculocutaneous Albinism 2 (*OCA2*) region*. For this region, we sequenced exons and promoters of the genes *OCA2* and *HERC2*, both of which have been previously associated with melanoma development. The initial individual association tests identified two SNPs associated with melanoma (**Supplementary Figure 1a**, **Supplementary Table 1**), both in one haplotype with lead SNP rs12593929, falling on an intron in *HERC2*. The most strongly associated SNP after the first logistic regression, in a separate haplotype, was rs62001328, that had an initial association *P* value < 0.03. The two SNPs in the first haplotype were associated with a range of pigmentation traits (**Supplementary Figure 1b**), as expected, whereas the SNP leading the second haplotype could not be imputed in the UKBiobank data and therefore association scores could not be calculated. However, no association was found between this SNP and the one described as the lead SNP in Landi *et al* (4) (rs12913832). No SNPs in this region were found associated with MSS.

*E-cadherin (CDH1) region*. We sequenced the promoters and exons of two genes of interest (*CDH1* and *CDH3*) (**Table 1**). The initial association tests identified an intronic variant within *CDH1*, rs181642569, as associated with melanoma risk (**Supplementary Figure 2a, Supplementary Table 1**). The second most associated SNP after logistic regression was rs35789195, but no melanoma-linked phenotypes were found associated with these (**Supplementary Figure 2b**). However, both SNPs seem unlinked to the lead SNP from the meta-analysis identified by Landi *et al* 2020 (4) (rs4420522).

*Cyclin D1 (CCND1) region.* We sequenced exons, promoters and DHSs for four genes of interest: *CCND1, MYEOV, ORAOV1* and *FGF19* (**Table 1**)*.* The initial Fisher’s association tests identified a downstream SNP from *FGF19*, rs17136816, as associated with melanoma with *P*-value = 0.0008546 and OR of the reference allele of 1.836 (**Supplementary Table 1**, **Supplementary Figure 3a**). However, it is not associated with the lead SNP identified in Landi *et al* in this region (rs4354713). The next most highly associated SNP was rs77925438, also not correlated with rs4354713. No melanoma-linked traits were found associated with any of these two SNPs (**Supplementary Figure 3b**).

**Analyses for population stratification in this cohort**


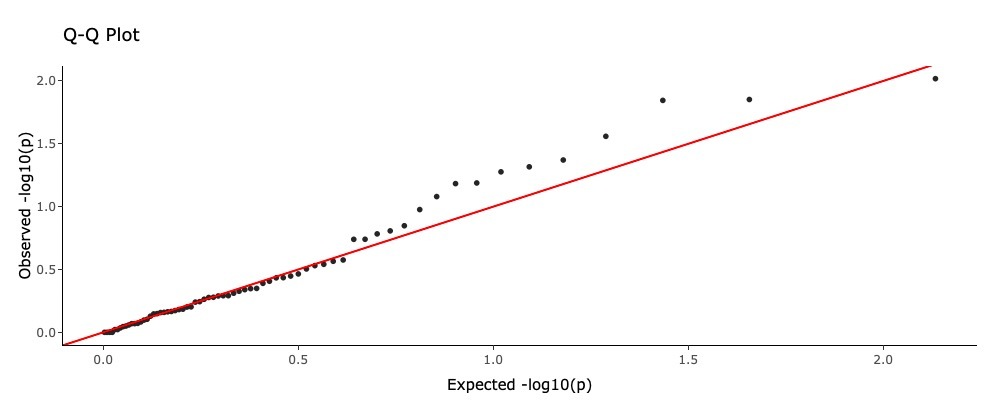


**QQ plot for common (MAF>0.01) synonymous variants in this study.** This plot was produced in order to investigate whether genomic inflation was present in the full dataset (1,977 cases and 754 controls). The observed genomic inflation (λ=1.12) is minimal. If it had been present, it may reflect the fact that these regions were chosen because of their known association with melanoma risk.

Additionally, a principal component analysis of the final included dataset (1,959 cases and 737 controls) shows that all samples cluster with the CEU (Northern/Western) European samples, and not the southern European (Tuscan) samples, so are highly homogeneous even within Europe (**Supplementary Figure 4**).
